# Exploring stress management strategies among emergency medical service providers in Iran: a qualitative content analysis

**DOI:** 10.1186/s12873-024-01024-8

**Published:** 2024-06-26

**Authors:** Afshin Khazaei, Ali Afshari, Rasoul Salimi, Abbas Fattahi, Behzad Imani, Mohammad Torabi

**Affiliations:** 1 Department of Medical Emergencies, Asadabad School of Medical Sciences, Asadabad, Iran; 2 Nursing Department, School of Nursing and Midwifery, Chronic Diseases (Home Care) Research Center, Hamadan University of Medical Sciences, Hamadan, Iran; 3 Department of Emergency Medicine, School of Medicine, Besat Hospital, Hamadan University of Medical Sciences, Hamadan, Iran; 4 Department of Medical Library and Information Sciences, Hamadan University of Medical Sciences, Hamadan, Iran; 5 Department of Operating Room, School of Allied Medical Sciences, Urology and Nephrology Research Center, Hamadan University of Medical Sciences, Hamadan, Iran; 6 Department of Nursing, Malayer School of Nursing, Hamadan University of Medical Sciences, Hamadan, Iran

**Keywords:** Stress disorders, Coping strategies, Emergency medical service, Qualitative research

## Abstract

**Background:**

Emergency medical service providers are frequently exposed to a variety of stressors as a result of their work environment. These stressors can have detrimental effects on both the physical and mental well-being of individuals. This study was conducted with the aim of exploring stress management strategies in emergency medical service providers.

**Methods:**

This study was conducted in 2023 using a qualitative approach and content analysis method. A purposive sampling method was used to include 16 emergency medical system providers from Hamadan city. Semi-structured interviews, with a duration of 45–60 min, were conducted for data collection. The Data were analyzed using Graneheim and Lundman’s conventional content analysis approach.

**Results:**

The analysis of the interview data revealed three themes: readiness for the worst conditions, assistance based on supportive partnerships, and striving for balance. The six categories within these three themes were mental preparation, risk management, collaborations in emergency response, supportive communication, adaptive behaviors, and maladaptive responses.

**Conclusions:**

The results of this study shed light on the various stress management strategies employed by emergency medical service providers. Understanding and implementing effective stress management strategies can not only enhance the well-being of emergency medical service providers but also improve the quality of patient care. Further research and action are essential to promote the resilience and mental health of these professionals, ensuring their overall well-being and job satisfaction.

**Supplementary Information:**

The online version contains supplementary material available at 10.1186/s12873-024-01024-8.

## Introduction

Workplace stress refers to emotional, cognitive, behavioral, and physiological responses to adverse aspects of work, the work environment, and the work organization. These reactions can lead to negative psychological and physiological consequences for the individual [1]. The prehospital emergency setting is one of the most stressful areas of the healthcare system. Emergency medical service (EMS) providers, as frontline personnel providing emergency services in this setting, are exposed to numerous stressors. These stressors include exposure to accidents and harrowing scenes, as well as the need to make quick decisions to rescue the injured [2–4]. Some of the stress factors that EMS providers face include rescuing patients in critical condition, caring for severely ill patients, facing the death of patients, excessive expectations from people, lack of organizational support, being exposed to violence and threatening situations at the scene of an accident, and the risk of contracting diseases from patients and injured individuals [5, 6]. Due to exposure to multiple stress factors, especially traumatic and critical incidents, EMS providers experience various health-related issues [7].

Ignoring workplace stress and neglecting prevention methods and stress reduction strategies in EMS providers can lead to various physical and psychological complications, such as anxiety, depression, sleep disorders, fatigue, digestive symptoms, unsafe behaviors, and job burnout [8]. It can also cause behavioral problems, such as a tendency to consume alcohol and cigarettes, and to quit and leave the service, ultimately leading to a lack of human resources and imposing extra costs on health systems [9]. Studies show that continuous exposure to work-related stress factors and neglecting stress reduction mechanisms in EMS providers are predisposing factors for posttraumatic stress disorder (PTSD) [10, 11].

Sterud et al. believe that EMS providers are reluctant to seek help from psychologists or doctors [6]. Some studies have shown that when nurses do not have enough time to adapt or reduce stress after experiencing stressful incidents, it can have detrimental effects on their well-being [12, 13]. Therefore, considering the nature of the prehospital field, it can be inferred that EMS providers will also be affected by the consequences of stress. This is due to being assigned multiple and complex missions without sufficient time for recovery [14].

Previous studies have reported that the level of stress among EMS providers in Iran is moderate to severe [15, 16] However, coping and adapting strategies for stress in this group of people have not been investigated. On the other hand, there have been limited qualitative studies conducted on the mechanisms of stress adaptation among EMS providers. For several reasons, we used a qualitative method to investigate stress measurement strategies in this study. Qualitative methods enable a more in-depth exploration of individuals’ experiences, perceptions, and behaviors related to stress management. Qualitative research enables the collection of rich, detailed data that can provide insights into the complexities of stress management. Qualitative research allows for flexibility in data collection, enabling researchers to adapt their approach based on emerging themes and insights. Stress management is influenced by various contextual factors, such as cultural, social, and environmental factors. Qualitative methods are well suited for capturing these contextual nuances, providing a more holistic understanding of stress management practices within various populations and settings. The aim of this study was to explore the perceptions and experiences of EMS providers regarding stress management strategies in response to work-related stress factors.

## Method

In the present study, a qualitative research approach and the content analysis method were used. Qualitative research gathers participants’ experiences, perceptions, and behavior. It answers how and whys instead of how many or how much. It could be structured as a stand-alone study, relying purely on qualitative data, or it could be part of mixed-methods research that combines qualitative and quantitative data [17]. Content analysis is a method of qualitative research used to analyze information to gain a better understanding of the subject being studied [18]. In this study, the theoretical framework that guides qualitative research was social constructionism. This framework emphasizes the role of social and cultural factors in shaping individuals’ perceptions and experiences.

### Participants

The research took place in the city of Hamadan, which is located in western Iran, from November 2022 to July 2023. According to the most recent statistics from December 2022, Hamadan has a population of 678,318. According to the guidelines of the Ministry of Health in Iran, cities with a population of 50,000 people should have at least one ambulance base. For every additional 60,000 people above the base population of 50,000 people, an additional ambulance base is needed. According to national standards, there should be one ambulance base every 30 km along roads. Currently, there are 12 urban ambulance bases in Hamadan, which are considered sufficient and in line with population standards. There are eight road bases in Hamadan, each located 30 km apart, according to national standards. However, the coverage of rural roads is not satisfactory, as only approximately 40% of rural areas are within a 30-kilometer radius of road ambulance bases [19]. Compared to other cities in Iran, Hamadan is considered a medium-sized city, and the number of existing ambulance bases currently complies with national standards. Unfortunately, there are no national or local guidelines for managing work-related stress in the emergency medical system in Hamadan. This is particularly concerning, especially considering that this issue became more noticeable during the COVID-19 pandemic. Furthermore, appropriate measures were not taken to manage stress, replace injured personnel, or support the staff.

The EMS in Iran is a division of the Ministry of Health, Treatment, and Medical Education. The government funds EMS delivery, which is provided free of charge. Bases are staffed with a combination of emergency medical technicians and nurses who are deployed together on missions. The dispatch center is responsible for diagnosing emergency cases and deploying operational personnel to accident scenes. The dispatch center is staffed by experienced nurses and physicians with over 5 years of experience. As soon as the personnel at the dispatch center receive the call and determine that the incident is an emergency, they transmit the received information wirelessly to the nearest base located near the incident site. During the mission, additional information such as the patient’s condition, the accident scene, and access routes to the scene are transferred to the EMS providers if needed. The dispatch center monitors the dispatch of ambulances moment by moment and responds to EMS providers’ requests during missions. This follow-up by the dispatch center personnel will continue until the mission is completed and the EMS providers return to the base. The dispatch center records the time it takes for EMS providers to prepare for their next missions in the system. Each ambulance station in Hamadan city is strategically located to cover a specific area of the city. The average response time in the city is 8 min, while for road accidents, it is 14 min. The comfort facilities in the ambulance stations are adequate, and all of them are equipped with sports equipment for the physical preparation of EMS providers. In Hamadan city, emergency medical personnel work in rotations, spending six months at road bases and the next six months at urban bases. The training of these personnel is overseen by the medical emergency system training unit, which organizes annual in-service training courses for the staff. At each ambulance base, two EMS providers provide emergency services during a 24-hour shift. EMS providers are in contact with physicians at the dispatch center by phone for consultation during critical and complex situations. The operation of the EMS in Iran is independent of other emergency and security systems (the Red Crescent, fire department and police).

In this study, all the ambulance bases in Hamadan city (12 urban and 8 road bases) were considered for selecting the participants. Since working in the emergency medical system is always accompanied by various factors such as encountering unfortunate incidents, dealing with environmental hazards, and providing assistance under unpredictable conditions, all EMS providers in this profession are exposed to occupational stress. Therefore, in this study, despite employing a purposeful sampling method, an effort was made to incorporate the maximum variation technique by considering age, work experience, ambulance station location, and educational degree. All operational personnel of the emergency medical system who were working in road and urban bases were potential participants in this study. To conduct the interviews, we visited the emergency center and obtained permission from the management of the emergency medical system. We also explained the purpose of the study. The first person interviewed had 14 years of work experience. The next participant was selected for interviews via snowball sampling among the EMS providers. Since the interviewer did not have a previous relationship with the participants, he used snowball sampling to select the participants. After interviewing the EMS providers, they were asked to identify individuals who had experienced more stressful factors in the work environment to participate in the study. The criteria for participating in the study included having at least two years of work experience in ambulance bases and a willingness to participate. In the emergency medical system of Hamadan city, there are 146 EMS providers. A total of sixteen EMS providers were invited to participate in the study. It is noteworthy that all selected participants agreed to participate in the study, and none declined or withdrew their participation.

### Data collection

The semi-structured interview guide was created based on the research objectives, taking into account the input of all members of the research team. The interview guide was given to two psychology experts outside of the research team for review. At this stage, some questions were revised and modified. Two pilot interviews were conducted to ensure the accuracy of the interviews and to validate the interview guide. All authors evaluated the integrity and accuracy of these two interviews, and the final interview guide was prepared. Pilot interviews were not included in the final analysis. The data were collected by the corresponding author through individual, face-to-face, and semi-structured interviews. The interviewer, who holds a PhD in nursing and is currently a member of the Faculty of Nursing and Midwifery, had the requisite qualifications to lead this qualitative study. These qualifications encompassed extensive experience in designing, executing, and interpreting qualitative research projects, along with expertise in utilizing diverse qualitative research methods such as interviews, observations, and content analysis. Furthermore, the interviewer had undergone formal education or training in qualitative research through courses, workshops, or hands-on experience, and exhibited strong communication skills. Additionally, the interviewer has ten years of nursing experience and approximately 8 years of experience as a university faculty member. Currently, they are actively involved in multiple research projects as executives and collaborators. The interviewer did not have a previous relationship with the participants. The interviews were conducted outside of work shifts and with prior coordination with the EMS providers. The duration of each interview averaged between 45 and 60 min. The interviews were conducted in the Persian language. The interviews were conducted in a quiet environment. The interviews were conducted according to the guide and semi-structured questions (Table 1).

**Table 1:** Semi-structured interview guide

**Stage 1 Introduction:**
- Introduce yourself and explain the purpose of the study, informing the participant about the duration of the interview.
- Obtain informed consent from the participant.
- Assure the participant of the confidentiality of the information.
**Stage 2 Conducting the Interview**:
Background Information:
- Start by requesting the participant to provide explanations and information about their role as a provider of emergency medical services.
- Ask the participant to share their experiences during the years they have worked in the emergency medical system.
Stressful Factors:
- Request the participant to discuss the most common stressful factors they encounter in their role as an emergency medical service provider.
- Ask participants to talk about their understanding of stress and its impact on their work, personal, and social life.
Stress Management:
- Ask the participant to describe strategies they use to manage work-related stress.
- Based on participants’ responses, examine individual coping strategies (such as exercise, mindfulness) and organizational-level strategies (such as peer support).
- Question participants about the effectiveness of these strategies and any challenges they face in implementing them.
- Request the participant to discuss support systems within their organization or outside of their workplace when dealing with stressful missions.
Personal Experiences:
- Ask the participant to describe a specific incident or situation that was particularly stressful for them.
- Inquire how they managed that situation and what coping strategies they utilized.
**Stage 3 Closing**:
- Thank the participant for their time and valuable insights.
- Provide contact information for any follow-up questions or concerns.

Each interview began with general questions, such as “Can you talk about your roles and responsibilities as an EMS provider in the workplace?” “Please explain to me some of the missions that have caused or exacerbated stress for you,” “Could you please tell me about the methods you employ to mitigate the impact of work-related stress?” “Please discuss support programs and protocols for personnel who experience highly stressful incidents in the workplace.” Then, based on the participants’ answers, several follow-up questions were asked to clarify the information provided. Each interview was recorded with an MP3 player and then transcribed verbatim by the corresponding author.

### Data analysis

To analyze the data, the conventional content analysis method suggested by Granheim and Lundman was used. This method consists of five steps: transcribing the entire interview immediately after conducting it, reading the interview text to gain a general understanding of its content, determining the meaning units and initial codes, classifying similar initial codes into broader categories, and identifying the latent content in the data [20]. After each interview, the audio files were transcribed verbatim. The typed text was read several times to extract the meaning units. The typed interview text, along with the extracted meaning units, was provided to the participants two days after the interview. This allowed them to confirm or modify the initial codes. Some of the meaning units were modified during this process. After extracting meaning units from each interview and conducting member checking with the participants, the interview with the next participant was conducted. The meaning units were constantly compared, and the relationships between the concepts were identified. The initial codes were classified based on their similarities and differences. Data collection continued until saturation, which was achieved after twelve interviews [21]. Data saturation is commonly employed in qualitative research methodologies such as interviews, focus groups, content analysis, or ethnographic studies. During the data analysis phase, researchers assess whether they have reached data saturation by examining whether new data continue to provide unique insights or if it repeats information already collected. Four additional interviews were conducted to ensure data saturation. In this study, due to the transparency and clarity of the initial codes obtained from the interviews, repeated interviews of the participants were not conducted. Therefore, the data collection ended after 16 interviews were conducted with 16 EMS providers. In the next step, categories with similar meanings were merged into distinct groups. Finally, the main themes were extracted based on their meanings and the relationships among the categories. Some of the participants’ expressions and sentences were translated into English based on the extracted themes and are presented in this article. The collected data were analyzed in MAXQDA v. 2010. The research team consisted of five academic staff members: four individuals with PhD degrees in nursing, one individual with a specialist degree in emergency medicine, and one person holding a master’s degree in Medical Library and Information Sciences. The research team consisted entirely of men aged between 35 and 48 years. All members of the research team have experience conducting both quantitative and qualitative research.

### Rigor

Credibility was ensured through the researchers’ prolonged engagement with the data and member checking. Member checking involves going back to the participants of a study to verify the accuracy and interpretability of the data collected from them. This process allows participants to review the researcher’s findings, analysis, and interpretations to ensure that they accurately reflect their experiences and perspectives. Member checking helps to establish the credibility and trustworthiness of the research by confirming that the researcher’s interpretations align with the participants’ views. Confirmability was ensured through peer checking. Peer checking involves seeking feedback from colleagues, experts, or other researchers in the field to review and critique the research process, methodology, analysis, and findings. Peers provide an external perspective on the research study, offering insights, suggestions, and critiques that can help strengthen the quality and rigor of the research. In the interviews, the generated codes and categories were shared with two external experts in the field of qualitative studies to confirm and validate the coding process. Before analyzing the data, a meeting was held between the research team regarding the stages and coding process. Additionally, to ensure dependability, three members of the study team, all of whom had extensive experience in qualitative studies, independently conducted all encoding stages. They subsequently reached a consensus after participating in multiple sessions to discuss the findings of the analysis. In these sessions, according to the research objectives and interview questions, some primary codes and subcategories were modified. To ensure transferability, various methods were employed, including sampling with maximum variation, providing detailed descriptions of participants’ characteristics, and comparing the study findings with those of other studies.

### Ethical considerations

This study was approved by the Institutional Review Board and the Ethics Committee of Hamadan University of Medical Sciences, Hamadan, Iran. (IR.UMSHA.REC.1401.392). Before participating in the study, participants were provided with detailed information about the research purpose and benefits. Participants were informed that participation in the study was entirely voluntary and that they could withdraw from the study at any time. The participants were asked to sign an informed consent form acknowledging their understanding and agreement to participate. To protect the identity of the participants, the researchers assigned codes to each participant in the study. All data collected during interviews, such as audio recordings, transcripts, and field notes, were securely stored and accessible only to the research team. Finally, the institution’s ethics committee ensured that the research protocols adequately protected participant confidentiality and adhered to ethical standards.

## Results

A total of sixteen EMS providers were asked to participate in the study. All participants engaged in regular exercise during the week, utilizing the sports equipment available at the bases. Among the participants, only two reported smoking (only after being in stressful situations and experiencing insomnia). Additionally, four participants exhibited irregular sleeping patterns, with an average of 12 sick days per year recorded for this group. The other demographic characteristics of the participants are shown in Table 2. Analyses of the data obtained from the interviews revealed three themes and six categories (Table 3).

**Table 2:** Characteristics of the participants

Characteristics of Participants	*N* (%) or Range (Median)
Educational degree	
EMT	3(19)
BS in EMS	6(37)
BS in Nursing	5(31)
MSc in Nursing	2(13)
Years in Practice	3–26(14)
Age	25–49(36)
Gender	
Male	16(100)
Marital Status	
Married	13(81)
Single	3(19)
Ambulance station location	
Urban	11(69)
Rural	5(31)

**Table 3:** Themes and categories extracted from the content analysis

Theme	Category	Subcategory
Readiness for the worst conditions	Mental preparation	Mental review of upcoming actions
		Situation-based information
	Risk management	Safe driving
		Incident scene management
		Self-protection
Assistance based on supportive partnerships	Collaborations in Emergency Response	intra organizational cooperation
		Interorganizational interactions
	Supportive communication	Interpersonal communication
		Stress Relief Consultation
Striving for balance	Adaptive behaviors	Distraction
		Good habits
		Spirituality
	Maladaptive responses	Inappropriate behaviors
		Substance abuse

Description of the coding tree: Three main themes were obtained from the data analysis in this study. One of the main themes was related to the stage before exposure to the stressful factors and conditions of the work environment and various types of accidents in the prehospital field. This theme was obtained from the two main categories of mental preparation and risk management. This theme was called “Readiness for the worst conditions”. The second theme was related to seeking help and consulting from others in the early stages after facing stressful factors. This theme was obtained from the two main categories of collaborations in emergency response and supportive communication. The third theme, “Striving for Balance “, was related to the behaviors and responses that the participants used as strategies and mechanisms to adapt to stress. This theme was obtained from two main categories of adaptive behaviors and maladaptive responses.

### Readiness for the worst conditions

#### Mental preparation

Most missions to which the emergency medical system is dispatched are urgent. The more EMS providers possess the mental preparedness and focus necessary for rescue, along with adequate information about the conditions at the scene, the safer and less stressed they will be. The main category of mental preparation consists of two subcategories: “mental review of upcoming actions” and “Situation-based information.”

##### Mental review of upcoming actions

The participants mentioned that all the missions were stressful because of the unknown. However, based on the information we have received from the patient and the accident scene, we can reduce the stress of the missions to a certain extent by mentally imagining and going through the upcoming actions according to the patient’s condition.

*We were sent to a mission that the operator said was a young person who had hanged himself. On the way to the scene, I went over all the actions I might need to take for this person… I can work better at the scene. (P6)*

*Being sent to scenes that we have experienced many times before brings us less stressful conditions. Whenever we recall our actions in previous cases, the scene of the incident no longer seems unfamiliar. (P2)*

##### Situation-based information

Some EMS providers emphasized in interviews that staff members who possess more information, knowledge, and experience in emergency incident management will experience less stress when providing relief. Participants mentioned that before arriving at the accident scene, they received the necessary information about the incident through the ambulance radio and the dispatch center. This helps them to be more prepared.

*I usually have the habit of calling the dispatch center several times on the way to the scene to gather additional and new information from the patient and the scene of the accident… This helps me to know exactly what conditions I am going to face. (P8)*

*I used to work in a hospital, specifically in a cardiac care unit, for a few years… I have extensive experience in caring for cardiac patients… I handle any dispatch related to a cardiac patient without stress. (P11)*

#### Risk management

Participants in the present study expressed that work in the prehospital emergency field differs significantly from work in healthcare facilities and hospitals. During the interviews, they discussed the various risks they faced during the dispatch and rescue process. They emphasized the importance of being physically and mentally prepared for deployments. Additionally, the ability to identify and mitigate hazards at the incident scene can reduce stress during an emergency response. The main category of risk management consists of three subcategories: “safe driving”, “incident scene management " and “self-protection”.

##### Safe driving

One of the main duties of EMS providers is to drive the ambulance. Most of the EMS providers mentioned in the interviews that they always experience stress due to the high speed and the risk of accidents and collisions with other vehicles during missions. They emphasized that using sirens and alarms during dispatch actually adds to their stress levels.

*The first year I entered this job, I drove very fast… I used to always have a racing heartbeat when driving, but now I drive better and no longer experience those symptoms. (P2)*

*Ever since I got into an accident while driving an ambulance, I have been following all the safety tips for driving. The work environment is stressful enough. I try to avoid adding extra stress by driving safely. (P11).*

##### Incident scene management

The participants identified proper management of the accident scene as one of the important factors in reducing stress during missions. They emphasized that scene management ensures the safety of rescue personnel, improves the patient’s condition, and prevents secondary accidents at the scene.

*The important lesson we learned from our experienced colleagues is that if we fail to manage the incident scene effectively, we will encounter more trouble and complications. (P9)*

*Gasoline spilled from the tanker’s reservoir onto the road surface. Many people had gathered around the tanker. The driver sustained minor injuries, so we transferred him to the ambulance. We immediately directed the people and parked cars near the accident scene to a safe place. Our priority was to prevent fires and harm to others. (P8)*

##### Self-protection

Most of the participants discussed the importance of personal protection and safety for EMS providers, as it helps to reduce stress at the accident scene. They considered this issue to be of the highest priority in missions and emphasized the importance of ensuring safety at the scene. They emphasized the importance of taking certain measures, such as wearing personal protective equipment, using appropriate equipment, maintaining a safe distance from hazardous elements, and following the instructions provided by local authorities.

*Most of the mission environments are unknown, and our first priority during the mission is to eliminate or reduce environmental risks. If our health is endangered, we cannot take action for the patient either. (P10)*

*The risk of contracting diseases and infections during all missions is something that causes us stress. However, wearing masks, gloves, and protective glasses, can reduce some of this stress. (P4)*

### Assistance based on supportive partnerships

#### Collaborations in emergency responses

Collaborations in emergency responses in the EMS involve effective communication and coordination among team members. This includes sending and receiving information at the accident scene, exchanging ideas, planning, and making decisions regarding the treatment and transfer of patients, and interacting with other rescue, security, and support services, such as medical equipment and medications. The main category of collaborations in emergency response consists of two subcategories: “intra organizational cooperation” and “interorganizational interactions”.

##### Intra organizational cooperation

EMS providers discussed the high levels of stress associated with road accidents and multiple casualty incidents. They stated that in such situations, having the assistance and support of colleagues from nearby stations, as well as the presence of a physician at the accident scene, would alleviate their workload and stress.

*In some missions where an extra ambulance is required to transport multiple injured individuals, our colleagues are dispatched promptly and with the utmost proficiency to assist personnel at the accident site. This instills confidence in us and alleviates our stress*. (P5)

*The psychiatric patient was uncontrollable, aggressive, and threatening… He was strong and powerful… We were scared and anxious that he would hurt us… We radioed one of the nearby EMS stations… When our colleagues arrived, there were four of us, and together we were able to restrain the patient and administer a sedative drug… (P9)*.

##### Interorganizational interactions

Some of the missions are related to crime scenes. Some others are related to car accidents that require cutting the car body to free the injured. The participants stated that in such cases, they need to collaborate and seek assistance from agencies such as the police and fire department. The presence of these factors promotes peace of mind and reduces stress.

*The firefighters arrived earlier than us because they were close to the accident site. They extinguished the fire on the bus, allowing us to safely evacuate the injured passengers. (P13)*

*In my opinion, the worst missions are crime scenes. If police personnel are present at the scene during these missions and establish security, we can more easily carry out rescue and relief measures. (P4)*

#### Supportive communication

The EMS providers mentioned in interviews that discussing, conversing, and sharing experiences with colleagues regarding stressful incidents and scenes had an impact on reducing stress levels. Some participants stated that in several cases, the intensity of stress was so high that they sought psychiatric help. The main category of supportive communication consists of two subcategories: “interpersonal communication” and” stress relief consultation”.

##### Interpersonal communication

Most of the participants stated that they typically engage in conversations and discussions with their colleagues regarding what they observed at the accident scene after completing their missions. Others also mentioned that they sometimes share their feelings with the physician at the call center.

*After stressful missions, I consult with colleagues who have more experience in the emergency medical system… it is truly helpful. (P11)*

*The scene of the accident was chaotic… I had a disagreement with one of the patient’s relatives and was rude to him. I was worried that he would surely file a complaint against me… A few hours later, I discussed it with one of my coworkers over the phone… I felt much better… He told me not to stress about it. (P5)*

##### Stress relief consultation

Some EMS providers who had experience dealing with highly stressful situations, such as burns, explosions, and crush injuries, reported experiencing symptoms of posttraumatic stress disorder (PTSD) for several weeks after the incidents. They sought help from a psychiatrist and received drugs to alleviate symptoms of stress and anxiety.

*Sometimes the stress level is very high, and all my thoughts become entangled. Even on days when I’m not on duty, reflecting on past missions induces stress and anxiety… I need to consult with a psychologist during these times. (P9)*

### Striving for balance

#### Adaptive behaviors

Most participants in the interviews mentioned that they use various methods to reduce stress, especially after completing stressful missions. These methods include reading books, watching television, and engaging in sports at the base. They emphasized that these methods are helpful in managing work-related stress, concerns, and daily tensions. The main category of adaptive behavior consists of three subcategories: “distraction”, “good habits” and “spirituality”.

##### Distraction

Participants said that they sometimes distracted themselves and tried to reduce the stress of their shifts by listening to music or watching comedy movies.

*Listening to my favorite songs during my work shift, especially before going to bed, relaxes me to some extent and reduces the stress caused during the day. (P12)*

*After we return from the mission, we play with the PlayStation. This helps us temporarily forget the incidents and stresses that we have experienced. (P11)*

##### Good habits

Participants in the interviews mentioned that on days when they are not on a shift, they usually engage in regular physical activity. They stated that these activities are highly effective in reducing work-related stress levels. EMS providers referred to activities such as mountaineering, cycling, and traveling with family.

*Being in nature is truly relaxing. I try to take walks in the park with my spouse on days when the weather is good. Seeing flowers, trees, and children are very soothing. (P8)*

*On the days when I am not on a shift, I have a gym schedule with friends. Sports programs strengthen the body and mind. In our opinion, sports should be mandatory. (P3)*

##### Spirituality

In their talks, EMS providers referred to spirituality, faith in God and belief in the power of God and activities such as prayer and supplication. They said that spirituality can give hope and motivation to a person. This belief helps a person cope better with stress and worries.

*Although our work is very stressful, I believe that because we save lives, God will surely help us. God is watching our actions… I find calmness in remembering God. (P6)*

*Praying and worship are fundamental practices in our religion that have been upheld since ancient times. I believe in my heart that communicating with God can help eliminate many negative thoughts, anxiety, and stress. (P13)*

#### Maladaptive responses

A few participants mentioned using various methods to cope with workplace stress, including taking sedatives and anti-anxiety medications, smoking cigarettes, and engaging in unconventional behaviors such as aggression. The main category of maladaptive responses consists of two subcategories: “inappropriate behaviors “and” substance abuse.”

##### Inappropriate behaviors

Some participants mentioned that they sometimes exhibit undesirable behaviors to relieve stress. They mentioned behaviors such as aggression toward patients’ companions or colleagues in the work environment, being quiet and withdrawn in the home environment, and experiencing social isolation during the interviews.

*After the shift, when I get home, I don’t talk to my family most of the time … I know it’s not the right way to alleviate fear and anxiety, but it seems to have become a habit. (P9)*

*Sometimes, the stress and pressure from one mission can manifest as verbal violence toward the patient or their relatives in the next mission. (P9)*

##### Substance abuse

Some participants mentioned that they sometimes have to use sleeping pills, tranquilizers, and cigarettes. The participants were individuals who had a history of smoking and drug use a few years ago.

*Although I know that smoking is harmful, but in my opinion, it is a good sedative… (P7)*.

*Due to the intensity of work pressure and the stress of the work environment, I sometimes have to use sedatives or soporific drugs before going to sleep. (P9)*

## Discussion

This study aimed to explore stress management methods among EMS providers. The present study shows that participants employ various strategies to manage occupational stress. The findings revealed that these strategies can be classified into three themes: readiness for the worst conditions, assistance based on supportive partnerships, and striving for balance. These findings will be discussed further.

According to the interviews, EMS providers stated that working in the prehospital emergency field is very stressful. Stress among EMS providers is a global issue that can have a detrimental impact on their health, well-being, and quality of their service [22]. The primary factors contributing to work-related stress among EMS providers are related to the incident scene, performing emergency procedures, and stabilizing and transferring patients. The findings of the present study have shown that EMS providers strive to achieve sufficient mental and physical preparedness prior to encountering and arriving at the scene, to better cope with these factors. Given the unfamiliar and uncertain conditions of the scene, as well as the uncertainty of the information received from individuals present at the incident, mental and physical preparedness can increase self-confidence during missions and reduce stress caused by stressors [23]. This preparedness is typically based on an EMS provider’s previous experiences [24]. The more information related to the patient’s emergency conditions, the location of the incident, and the atmosphere of the scene that is received and transmitted to the EMS provider by the call center, the better equipped these individuals are to provide safe and timely services to the patients through proper scene management [25, 26]. On the other hand, improved management of the incident scene and strict adherence to safety precautions when dealing with hazardous factors can also guarantee their own security and safety [27]. Creating safe conditions at the incident scene reduces the severity of work-related stress for these individuals [7]. EMS provider, by considering the worst possible conditions at the scene and mentally imagining the patient’s condition, can reduce stress by being mentally and physically prepared, making emergency equipment available, and timely calling other emergency and security personnel [28]. Mental readiness is a complex structure that includes dimensions such as attention control, goal setting, calmness, activation, self-confidence, self-talk, and visualization [29–31]. Marquardt et al. showed that mental preparedness in occupations that involve emergency responses, such as firefighters and EMS providers, can reduce stress and enhance performance and success [32]. Bohstrom et al., who investigated stress management methods among ambulance nurses, reported that nurses reduce their stress by receiving accurate and complete information before being deployed on missions [14]. On the other hand, research has demonstrated that a significant factor contributing to stress in clinical and nursing professions is a lack of knowledge, awareness, and skills [33]. The results of these studies are consistent with the findings of the present study.

Another stress management strategy discussed by EMS providers in this study was risk management, which included three subcategories: safe driving, scene management, and self-protection. In Magaña’s study, which focused on risk factors in driving emergency missions, the driving process was divided into two categories: driver-related factors and environmental factors. In this study, it was suggested that safe driving and maintaining a proper longitudinal distance can help reduce driving risks [34]. In the present study, participants also employed methods such as practicing safe driving, maintaining a confident speed, and minimizing the use of sirens to reduce stress. Another stress management strategy among the participants was scene management. Afshari et al. showed that taking actions such as identifying environmental hazards, properly parking ambulances, stabilizing the scene, communicating information from the scene to the call center, triaging the injured, and estimating the number of personnel and equipment could prevent increased stress at the scene [7]. This result from the present study is not observed in other papers related to the emergency medical system. This can be attributed to the independence of the emergency medical system from other relief organizations in Iran. This has resulted in the EMS provider being the first responder in most cases of disasters and accidents. They are responsible for managing the accident scene. The coordination and summoning of other relief agencies is the responsibility of the EMS provider at the scene of the accident. Another stress-reducing factor among EMS providers is prioritizing personnel safety and using personal protective equipment during missions [35, 36]. EMS providers often have limited information about their patients, work under uncontrolled conditions, and accompany their patients in confined ambulance spaces [19, 37]. These individuals are at risk of contracting various infectious diseases. Therefore, implementing standard precautions and utilizing personal protective equipment will minimize the risk of transmitting infectious agents [38]. In a qualitative study conducted by Sanlıtürk, one of the stressors identified among nurses was the lack of personal protective equipment [39]. The issue of a lack of personal protective equipment (PPE) in Iran and in almost all countries, especially during the COVID − 19 pandemic, has been highlighted and is considered a significant source of stress in this profession [35].

Prehospital emergency system missions are carried out by only two personnel. However, in cases where the scene is crowded or there are multiple casualties, additional personnel are called in. A high workload, a low number of personnel at the scene, and a lack of cooperation and coordination among other relief organizations are stressful factors in the prehospital field [2]. Participants in this study indicated that consultation with colleagues on necessary actions for patients, teamwork at the scene, support from personnel and equipment from nearby bases, and the involvement of other aid and safety organizations such as the Red Crescent and Police in some operations provided peace of mind and reduced stress. Therefore, the involvement, cooperation, and support of colleagues and other organizations involved in rescue and relief operations have been identified as a solutions to prevent or reduce stress among EMS providers [2]. A study by Holmes et al. also showed that teamwork in the clinical setting reduces stress among personnel [40]. Another point mentioned by EMS providers for stress reduction was the support and understanding of colleagues and superiors for the stresses imposed on them. They said they mostly used the method of talking and conversing with colleagues or non-colleagues to express their feelings and reduce stress. Haus et al. also showed that organizational support and peer support play important roles in reducing mission-related stress [41]. In another qualitative study that examined stress coping strategies among EMS providers in 2015, peer support was identified in subgroups of informal conversations, expert and peer support, and informal clarification at the end of the shift [14], which is consistent with the findings of the present study. Some of the participants who had experience dealing with high-stress incidents indicated that the intensity of stress after the incident is so severe in some missions that there are signs of stress and anxiety for weeks, and psychologists should be consulted. Weber et al. showed in a study that psychological treatments by mental health professionals are effective and have been proven to treat PTSD [42]. In Iran, the relief systems, including the EMS and the Red Crescent Organization, as well as security and firefighting organizations, are distinct and operate independently from one another. In many incidents and missions, the presence of multiple rescue teams with various tasks is needed. On the other hand, EMS providers are usually the first to respond at the scene. Therefore, with the simultaneous or immediate presence of other security and relief organizations at the scene of the accident, the workload of EMS providers will be decrease, and they will experience less stress [7]. Currently, the emergency medical system in Hamadan city lacks specific protocols for identifying personnel experiencing stress-related issues and referring them to counseling centers. The results revealed that employees attempted to cope with work-related stress by seeking advice and talking to colleagues. In cases where these strategies were unsuccessful, they sought help from a psychiatrist without informing the emergency medical authorities.

Participants indicated that they use methods to reduce and eliminate stress after experiencing stressors related to emergency missions within the base and on days when they are not on duty. Most EMS providers cited methods such as distraction (reading books, listening to music, watching movies, and playing computer games), good habits (exercise, walking), and spiritual activities (prayer, praying, participating in religious ceremonies) to reduce stress. Fallon et al. showed that music can affect mood and physiological states. They demonstrated in an intervention study that listening to music reduced stress and enhanced mood [43]. A study by Janson and Rohleder showed that distraction, as opposed to denial, leads to early stress reduction and faster recovery in individuals experiencing stress [44]. Studies have shown that exercise and physical activity, such as walking, have significant positive effects on preventing or reducing mental illnesses, including symptoms of depression, anxiety-related disorders, and stress [45–47]. Considering the Islamic community in Iran and their religious beliefs, many EMS providers find that their emotional connection to God, performing religious commands, and going on pilgrimage trips are highly effective in reducing or eliminating work-related stress [48]. A study conducted on nurses in intensive care units showed that among the methods of coping with stress, belief in religion was the most common method, while substance abuse was the least common method of adaptation [49]. Another study also showed that emergency nurses primarily relied on positive spiritual coping as their main coping strategy, while demonstrating a lower inclination toward using negative spiritual coping strategies [50]. In many religions, spirituality and spiritual practices play a significant role in reducing stress and promoting adaptation. For example, visiting sacred places, participating in religious ceremonies, reciting scriptures and prayers, meditating, and focusing on God and faith in fate can help individuals cope better with life’s stresses [51, 52]. Furthermore, many religions recommend that individuals turn to God and their faith, finding hope in them. Believing in the existence of a greater and more powerful force behind everything can give individuals a sense of peace and hope, aiding in stress reduction [53]. In addition, religious communities often provide social support and a sense of belonging, which can help individuals overcome difficult times and cope better with stress [54]. However, there are also negative aspects to consider. Some religious teachings can cause feelings of guilt, anxiety or shame, which can increase stress and contribute to mental health problems. In some cases, rigid adherence to religious beliefs can lead to feelings of judgment, exclusion or isolation, which can exacerbate stress rather than alleviate stress [55]. Considering the presence of Islam and its religious teachings in Iran, the methods and strategies discussed in this article can be interpreted and justified.

In this study, a small number of participants mentioned behaviors such as aggression, social isolation, silence, and substance abuse as coping mechanisms for reducing stress. These individuals believed that such behaviors, particularly substance abuse, were not suitable or effective methods for managing and alleviating work-related stress. However, they did not actively seek other suitable stress management methods and were more focused on seeking temporary and immediate relief from stress. Chronic stress, especially when experienced over a prolonged period, can result in lasting and progressive alterations in the brain, rendering individuals more vulnerable to drug use and addiction. These changes, which weaken and strengthen specific areas of the brain, lead to emotional states, impaired executive control, and a strong craving for certain substances [56]. Substance abuse can often be a response to stress and an attempt to cope with difficult emotions and situations. However, substance abuse is not a healthy or effective way to reduce stress or adapt to it. In fact, it often exacerbates problems and can lead to further stress and negative consequences [57].

In recent years, several studies in the field of prehospital emergency care have focused on stress management methods for EMS providers. Rojas et al. showed that EMS providers utilize methods such as support, acceptance, humor, spirituality, and religion to alleviate work-related stress [58]. Almutairi and ElMahalli’s study demonstrated that the most common strategy used for stress management among EMS providers is talking to colleagues, taking leave after stressful incidents, and reflecting on the positive aspects of work [59]. Another study conducted in 2023 on stress management methods among EMS providers using qualitative methods revealed main themes including seeking social support, self-care practices, coping mechanisms, and finding meaning and purpose at work [60]. Bohström et al. who used a qualitative approach to medical emergency personnel, showed that the strategies employed to manage mission-related stress included receiving accurate information from the call center, discussing with colleagues, advancing teamwork collaboration, and short rests between missions [14]. Most of the findings from these studies are similar to the results of the present study. A new theme identified in the present study, not previously observed in similar studies in the prehospital field, is the readiness of EMS providers for the worst conditions, encompassing two categories: mental preparation and risk management. It appears that participants in this study utilized strategies and measures to reduce stress even before facing stressful work environment factors.

Since all EMS providers in the system are male and there are currently no female personnel working in the emergency medical system of Hamadan city, a limitation of this study was the lack of female participants. The lack of female participants limits the generalizability of the study’s findings to the broader population of EMS providers. The results may not be representative of the experiences and needs of male and female EMS personnel, which limits the applicability of the recommendations made in the study. Women in male-dominated professions such as the EMS may face gender-specific stressors and barriers that are not addressed in the study. Without their perspective, the study may overlook important factors that influence female EMS workers’ coping with stress. To address these limitations, future research should aim to include a diverse range of participants, including both male and female EMS providers, to ensure a more comprehensive and inclusive understanding of stress management strategies in the profession.

## Conclusion

The results of this study showed that the majority of dispatches in the EMS are stressed due to the unknown and unpredictable conditions of the accident scene. This stress is perceived by the EMS providers from the time of announcing the mission to delivering the patient to the treatment centers and even, in some cases, for several days after that. Participants used different strategies to prevent and reduce stress of missions. These strategies were categorized into three themes: readiness for the worst conditions, assistance and striving for balance. Due to the nature of rescue and relief operations, EMS providers used stress management methods as strategies to prevent the development and escalation of stress from the moment the mission was announced by the dispatch center. During the rescue, they attempted to control and reduce stress by managing the scene, taking timely action, and working as a team, and finally, after facing stressors, they used adaptive mechanisms to reduce or eliminate the effects of stress. The results highlight the need to obtain accurate information from the incident command center about the scene, scene management and safety, interaction with other responders, and individual and organizational support. Proper stress management and organizational support for EMS providers after stressful events can promote better adaptation and resilience to workplace stress. In addition, this can help improve the mental health of EMS providers and, consequently, enhance the quality of service delivery in the emergency medical system. It is suggested to investigate the effect of stress management training courses on the stress levels of EMS providers in future studies.

### Electronic supplementary material

Below is the link to the electronic supplementary material.

Supplementary Material 1

Supplementary Material 2

## Data Availability

The datasets used and/or analyzed during the current study are available from the corresponding author on reasonable request.

## Abbreviations

EMS: emergency medical services
