# Supplementary material for: Exploring stress management strategies among emergency medical service providers in Iran: a qualitative content analysis
Source: BMC Emerg Med. 2024 Jun 26;24:106. doi: 10.1186/s12873-024-01024-8 (PMC11209986; doi:10.1186/s12873-024-01024-8)
Supplement: Supplementary file 2 — Supplementary Material 2 [file 12873_2024_1024_MOESM2_ESM.docx]

**Semi-structured Interview Guide** (Exploring stress management strategies among emergency medical service providers: a qualitative content analysis)

**Stage 1 Introduction:**

- Introduce yourself and explain the purpose of the study, informing the participant about the duration of the interview.
- Obtain informed consent from the participant.
- Assure the participant of the confidentiality of the information.

**Stage 2 Conducting the Interview:**

Background Information:

- Start by requesting the participant to provide explanations and information about their role as a provider of emergency medical services.
- Ask the participant to share their experiences during the years they have worked in the emergency medical system.

Stressful Factors:

- Request the participant to discuss the most common stressful factors they encounter in their role as an emergency medical service provider.
- Ask participants to talk about their understanding of stress and its impact on their work, personal, and social life.

Stress Management:

- Ask the participant to describe strategies they use to manage work-related stress.
- Based on participants' responses, examine individual coping strategies (such as exercise, mindfulness) and organizational-level strategies (such as peer support, debriefing sessions).
- Question participants about the effectiveness of these strategies and any challenges they face in implementing them.
- Request the participant to discuss support systems within their organization or outside of their workplace when dealing with stressful missions.

Personal Experiences:

- Ask the participant to describe a specific incident or situation that was particularly stressful for them.
- Inquire how they managed that situation and what coping strategies they utilized.

**Stage 3 Closing:**

- Thank the participant for their time and valuable insights.
- Provide contact information for any follow-up questions or concerns.
